# Molecular Classification of Bladder Urothelial Carcinoma Using NanoString-Based Gene Expression Analysis

**DOI:** 10.3390/cancers13215500

**Published:** 2021-11-01

**Authors:** Antonio Lopez-Beltran, Ana Blanca, Alessia Cimadamore, Rajan Gogna, Rodolfo Montironi, Liang Cheng

**Affiliations:** 1 Department of Morphological Sciences, University of Cordoba Medical School, E-14004 Cordoba, Spain; 2 Maimonides Biomedical Research Institute of Cordoba, Department of Urology, University Hospital of Reina Sofia, E-14004 Cordoba, Spain; anamaria.blanca@imibic.org; 3 Institute of Pathological Anatomy and Histopathology, School of Medicine, Polytechnic University of the Marche Region, United Hospitals, 60126 Ancona, Italy; alessiacimadamore@gmail.com (A.C.); r.montironi@staff.univpm.it (R.M.); 4 Faculty of Health and Medical Sciences, BRIC-Biotech Research & Innovation Centre, University of Copenhagen, 2200 Copenhagen, Denmark; rajan.gogna@bric.ku.dk or; 5 Faculty of Health and Medical Sciences, Novo Nordisk Foundation Center for Stem Cell Biology, DanStem, University of Copenhagen, Copenhagen N, Denmark, 2200 Copenhagen, Denmark; 6 Departments of Pathology and Urology, Indiana University, School of Medicine, Indianapolis, IN 46202, USA; liang_cheng@yahoo.com

**Keywords:** bladder cancer, molecular taxonomy, molecular, classification, NanoString, luminal, basal

## Abstract

**Simple Summary:**

Our study aimed to apply a quantitative method based on mRNA counting as nCounter (NanoString Technologies, Inc). This method can obtain precise and accurate measures of RNA expression compared to RT-PCR, and which might represent an alternative to the NGS-genomic/transcriptomic profiling frequently used to generate molecular data in bladder cancer and provide clinically meaningful datasets for the molecular classification of bladder cancer. The current study generated a four-gene classifier, incorporating GATA3 and KRT20 (typically related to luminal molecular subtype) and KRT5 and KRT14 (typically related to basal molecular subtype). This methodology allowed us to explore differences in clinicopathologic parameters and potential sensitivities to ICI immunotherapy in a cohort series of 91 urothelial carcinomas of the bladder.

**Abstract:**

Molecular classification of bladder carcinoma is a relevant topic in modern bladder cancer oncology due to its potential to improve oncological outcomes. The available molecular classifications are generally based on transcriptomic profiles, generating highly diverse categories with limited correlation. Implementation of molecular classification in practice is typically limited due to the high complexity of the required technology, the elevated costs, and the limited availability of this technology worldwide. We have conducted a gene expression analysis using a four-gene panel related to luminal and basal subtypes in a series of 91 bladder cancer cases. NanoString-based gene expression analysis using typically luminal (GATA3+/KRT20+) and basal markers (KRT14+/KRT5+/GATA3low/-/KRT20low/-) classified urothelial bladder carcinoma samples as luminal, basal, and a third category (KRT14-/KRT5-/GATA3-/KRT20-), null/double negative (non-luminal/non-basal). These three categories were meaningful in terms of overall cancer-specific survival (*p* < 0.0001) or when classified as conventional urothelial carcinoma and variant histology urothelial carcinoma (*p* < 0.0001), NMIBC vs. MIBC (*p* < 0.001), or by AJCC stage category Ta (*p* = 0.0012) and T1 (*p* < 0.0001) but did not reach significance in T2-T4 (*p* = 0.563). PD-L1 expression (low vs. high) was also different according to molecular subtype, with high PD-L1 expression mostly seen in basal and null subtypes and carcinomas with variant histology (*p* = 0.002). Additionally, the luminal subtype was enriched in NMIBC with favorable cancer-specific survival (*p* < 0.0001). In contrast, basal and null subtypes resulted in aggressive MIBC tumors with shorter cancer-specific survival (*p* < 0.0001), some of which presented variant histology. In conclusion, a comprehensive evaluation of a gene classifier related to molecular taxonomy using NanoString technology is feasible. Therefore, it might represent an accessible and affordable tool in this rapidly expanding area of precision genomics.

## 1. Introduction

Bladder carcinoma has been traditionally classified as non–muscle-invasive bladder cancer (NMIBC), including Ta, T1, and urothelial carcinoma in situ, and muscle-invasive bladder cancer (MIBC), including T2-T4 disease [1,2,3]. About 70% of patients belong to the NMIBC category, a disease characterized by frequent tumor recurrence, limited tendency to progress, and high survival rate following guidelines recommended therapy; however, recent risk-stratified NMIBC categories show higher variability than initially thought regarding tumor progression [4,5,6,7,8].

In contrast, patients with MIBC typically receive neoadjuvant chemotherapy (NAC) followed by radical cystectomy [9]. The locally advanced and metastatic disease typically requires biomarker-guided immune checkpoint inhibitors (ICI), targeted therapies, or other novel drugs conjugates [10,11,12,13,14,15,16,17,18,19].

In an attempt to better define urothelial carcinoma, molecular classification of these tumors might provide meaningful information to stratify prognostically relevant categories or to define the proper treatment in a given patient [13,20]. In this clinical scenario, the development of the molecular taxonomy of bladder cancer probably represents the most fascinating and important novelty in decades. Furthermore, using complex methodologies, MIBC has been classified into two wide molecular subtypes, luminal and basal, following the categorization currently in use in breast cancer [21,22,23,24,25,26,27,28]. Reportedly, these two wide categories present differences in prognosis and sensitivity to the current therapies, with the basal subtype being more aggressive than luminal [22,25,27,29,30].

**Figure 1:**
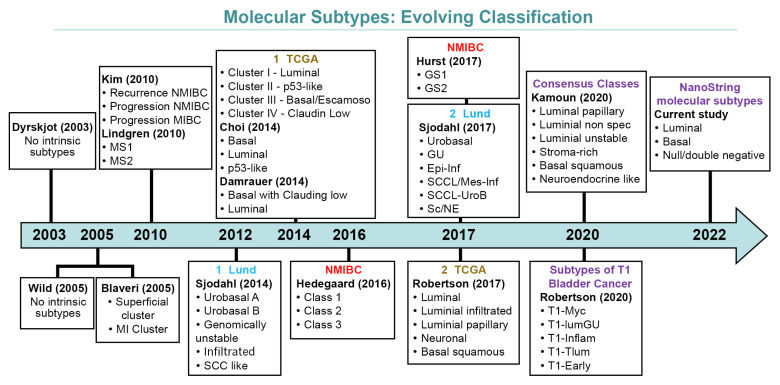
Evolving schemes of molecular classification of urothelial carcinoma of the bladder.

Differences in methodologies and the interpretation of earlier data resulted in several molecularly defined classifications currently available [13,20,28,31,32,33,34,35,36,37,38]. Figure 1 depicts how molecular classifications of bladder cancer have evolved over the years.

Despite the recent consensus on some molecular subtypes, the application of molecular classification still requires complex, expensive, and not frequently available technology [34,39,40,41,42]. However, the recent introduction of the novel NanoString technology to analyze gene expression provides an alternative for molecular subtyping, with the potential advantage of lower cost per sample to analyze and produce accurate gene classifiers with clinical application [35,43]. Therefore, a study conducted to explore NanoString technology in the context of the molecular taxonomy of urothelial carcinoma would be relevant and helpful to provide less expensive and reproducible tools to investigate the molecular classification of urothelial bladder carcinoma.

Our study aimed to apply a quantitative method based on mRNA counting as nCounter (NanoString Technologies, Inc., Seattle, DC, USA). This method can obtain precise and accurate measurements of RNA expression compared to RT-PCR and might represent an alternative to the NGS-genomic/transcriptomic profiling frequently used to generate molecular data in bladder cancer and provide clinically meaningful datasets for the molecular classification of bladder cancer. The current study generated a four-gene classifier, incorporating GATA3 and KRT20 (typically related to luminal molecular subtype) and KRT5 and KRT14 (typically related to basal molecular subtype). This methodology allowed us to explore differences in clinicopathological parameters and potential sensitivities to ICI immunotherapy in a cohort series of 91 urothelial carcinomas of the bladder.

## 2. Materials and Methods

### 2.1. Tumor Samples

In this study, we analyzed a retrospective cohort of cases that were collected from patients that underwent transurethral resection of bladder tumor between 2005 and 2014 at Reina Sofia University Hospital, Cordoba, Spain. Only patients with a primary diagnosis of MIBC or NMIBC bladder carcinoma, and no previous therapy other than the surgical procedure were included in the study series. After the surgical procedure, samples were immediately divided into two halves, one was snap-frozen and stored at –80 °C until processing, and the second one was formalin-fixed and paraffin-embedded. Histological evaluation was done on hematoxylin and eosin–stained glass slides. An experienced pathologist (ALB) classified, graded, and assessed the pathologic stage of each case following the 2016 WHO (World Health Organization, Geneva, Switzerland) classification of urologic tumors and the 8th edition of the AJCC (American Joint Committee for Cancer) [2,44]. Tumors classified as NMIBC were additionally stratified according to risk categories (low, intermediate, high and very high) [7,8].

A total of 91 samples were chosen for the current study after excluding 16 samples due to poor quality, patient loss to follow-up, or limited tumor volume present. All selected cases yielded adequate tumor volume and high-quality total RNA suitable for NanoString technology. Informed consent was obtained from all patients, and the study was approved by the Local Ethical Committee (Act #274-ref 3800/2018).

The number of months from the date of the surgical procedure to the date of the latest cystoscopy (or the last visit or death) defined the patient’s follow-up. The survival time was defined as the period between the diagnosis and death, and cancer-related death was caused by bladder carcinoma.

### 2.2. NanoStringcodeset Design

The mRNA expression levels of the four markers, GATA3 and KRT20, typically used to define luminal molecular subtype, and KRT5 and KRT14, typically used to define basal subtype, were considered the gold standard for molecular classification in the current study. Custom NanoString probes were designed to match the four classifier gene signatures. A verification set of five housekeeping genes (TBP, TUBA1B, ALAS1, ACTB, and SDHA) was selected based on their low coefficients of variance. The probe set verification was carried out using NanoString’s standard custom codesets, consumables, and assay procedures.

### 2.3. RNA Isolation and Quantification

Total RNA was extracted from pulverized bladder tumor tissue using RNeasy Mini Kit (Qiagen Inc., Valencia, CA, USA) according to the manufacturer’s protocol. RNA concentrations were assessed by spectrophotometry (NanoDrop; Thermo Fisher Scientific, Waltham, MA, USA) and re-assessed by BioAnalyzer (Agilent Technologies, Inc., Santa Clara, CA, USA). RNA quality was measured by the RNA integrity number and by the percentage of RNA fragments >100–300 nucleotides in size (DV100–300). Across high-quality samples, a minimum of 80% of RNA fragments >100 nucleotides (DV100 > 80) were included in our study.

### 2.4. Molecular Classification Based on NanoString Analysis

Transcripts were counted using the automated NanoStringnCounter system (NanoString Technologies, Seattle, WA, USA). Counts were normalized with the nSolver Analysis Software (version 4.0) with the Advanced Analysis (module 2.0.115) plugin. Raw counts data were normalized to internal positive control probes and housekeeping genes using background thresholding with a threshold count value of 20.

For the molecular classification, bladder urothelial carcinoma samples with high KRT20or GATA3 (GATA3+ or KRT20+) expression were considered luminal, high KRT5 or KRT14 expression and low-to-negative expression of luminal markers (KRT14+ or KRT5+/GATA3low/-/KRT20low/-) defined the basal subtype, and a third category with no expression of the four genes (KRT14-/KRT5-/GATA3-/KRT20-) was classified as null/double-negative (non-luminal/non-basal). We have observed rare GATA3+ and KRT20+/KRT14low/KRT5low cases also considered within the luminal subtype. Immunohistochemistry using antibodies against GATA 3, CK20, CK5/6, and CK14 was used as an additional internal control of the reaction.

### 2.5. PD-L1 mRNA Quantification by RT-qPCR

SYBR Green quantitative RT-PCR was applied to quantitate PD-L1 and the housekeeping gene RPS23 (ribosomal protein S23) expression. Each patient sample was analyzed in duplicate. Forty amplification cycles were applied, and the cycle quantification threshold (Ct) values of PD-L1 and RPS23 for each sample were estimated as the mean of the two measurements. Ct values were normalized by subtracting the Ct value of the housekeeping gene RPS23 from the Ct value of the target gene (ΔCt). Expression results were then reported as 40-ΔCq.

### 2.6. Statistical Analysis

All statistical analyses were performed with SPSS 25.0 (SPSS Inc, Chicago, Illinois) and MedCalc Statistical Software version 17.6 (MedCalc Software bvba, Ostend, Belgium). Patient and clinical characteristics were summarized as numbers and percentages. Normalized data were generated using the nSolver Analysis Software, and Metaboanalyst was used to generate the heatmaps, which were mean-centered and divided by the SD of each variable (scaled Z-score) [45]. Hierarchical clustering of RNA expression was performed using Euclidean distances and the Ward algorithm. The differentially expressed classifications of genes were dichotomized using the median and the receiver operating characteristic curve (Youden index) to determine the best cutoff point that allowed optimal separation between high versus low PD-L1 expression with maximum combined sensitivity and specificity. Survival analysis for cancer-specific survival (CSS) was carried out by Kaplan–Meier curves and compared by the log-rank test. Univariate and multivariate analyses were performed using Cox proportional hazards model. A *p*-value ≤0.05 was considered statistically significant.

## 3. Results

**Figure 2:**
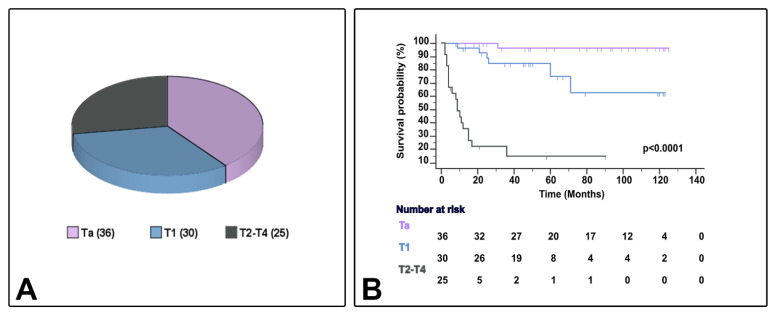
Sample distribution (**A**) and survival differences (**B**) by AJCC/TNM stage category in the current series.

Table 1 presents the characteristics of the 91 cases of bladder urothelial carcinoma with conventional urothelial morphology or variant histology (24 cases [26.4%]), including micropapillary (6.6%), nested (6.6%), plasmacytoid (5.5%), or other variants (7.7%) (squamous [3] or trophoblastic [1] divergent differentiation, giant cell carcinoma [2], and lymphoepithelioma-like carcinoma [1]). Eleven patients were female (12.1%), and the median age ranged from 45–95 years. In the current series, the AJCC stage category included Ta (39.6%), T1 (32.9%), and T2–4 (27.5%). Patient follow-up and survival status ranged from 2–125 months and 8–125 months, respectively, and CSS ranged from 2–71 months (Figure 2). Patients whose tumors were classified as NMIBC received BCG therapy (bacillus Calmete–Guerin) with maintenance according to validated guidelines at the time of diagnosis. In the current study, patients with MIBC did not receive neoadjuvant chemotherapy.

**Figure 3:**
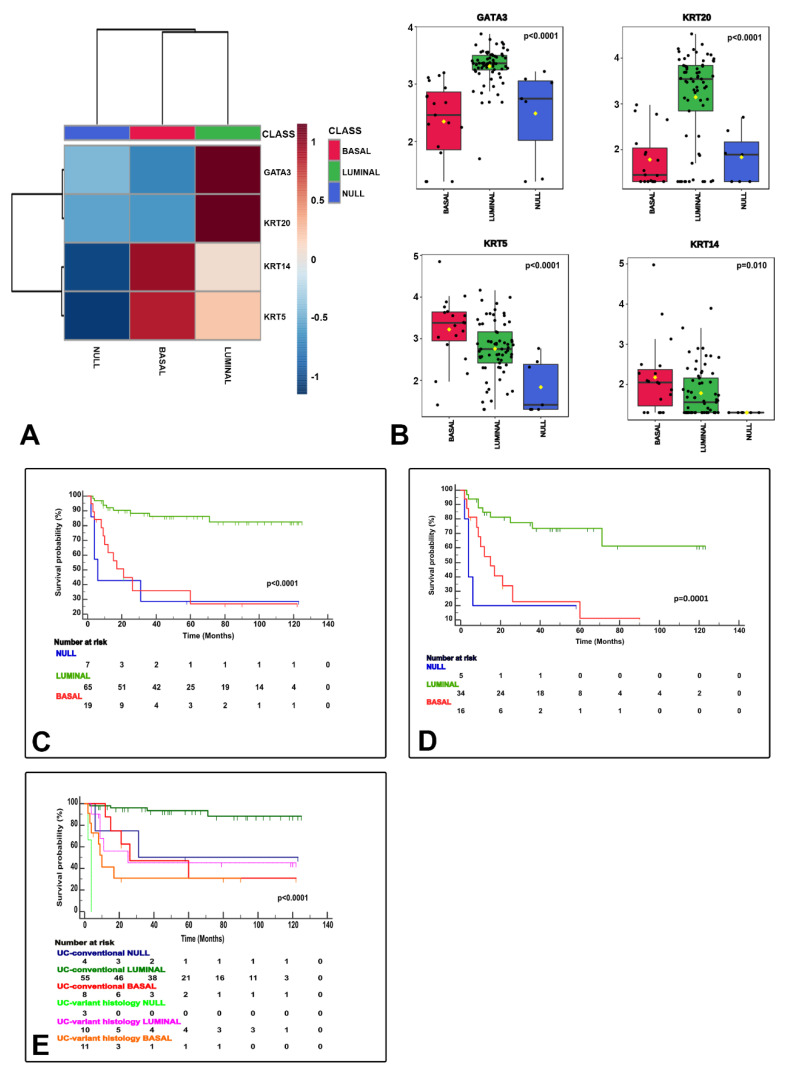
NanoString gene expression generated molecular classification of bladder cancer. The heatmap shows the luminal (GATA3+ and/or KRT20+), basal (KRT14+/KRT5+/GATA3low/-/KRT20low/-), and null (GATA3−, KRT20−, KRT5−, KRT14−) subtypes (**A**). Box and whisker plots of the normalized values (mean ± SD), illustrate the expression of GATA3, KRT20, KRT5, and KRT14 (**B**). The Kaplan–Meier plots identify meaningful molecular subtypes for CSS with luminal subtype as the less aggressive and basal/null-double negative subtypes being the more aggressive end of the spectrum (**C**). A subsequent substudy of “C” excluding stage Ta tumors is presented in (**D**). Molecular subtypes also expressed differences according to pathologic tumor classification (conventional vs. variant histology urothelial carcinoma) (**E**).

**Figure 4:**
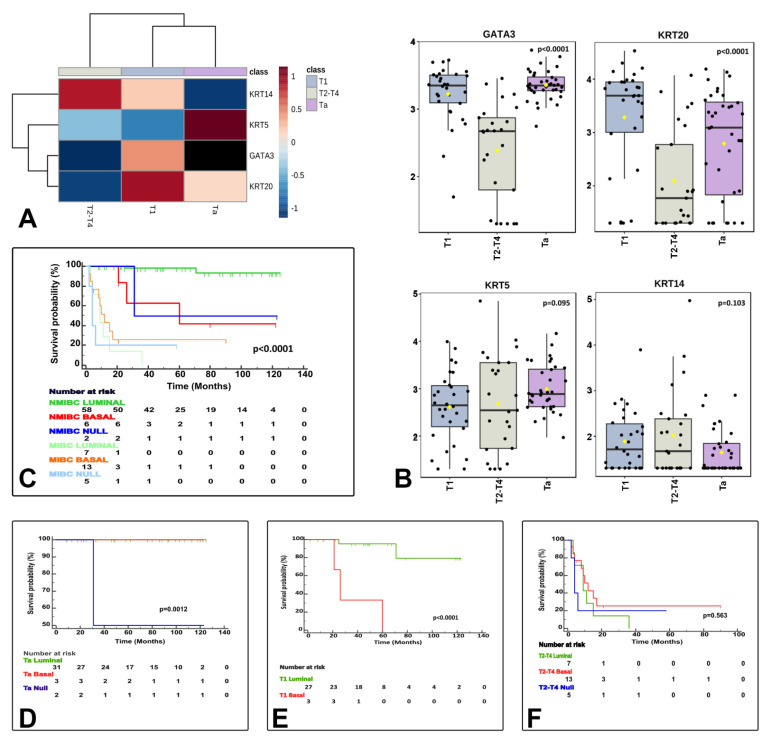
The three identified molecular subtypes correlate with tumor stage related features. The heatmap shows differentially expressed luminal and basal markers by stage category (**A**). Box and whisker plots of the normalized values (mean ± SD) illustrate the expression of GATA3, KRT20, KRT5, and KRT14 by stage category (ANOVA test) (**B**). The Kaplan–Meier plots identified differences for cancer specific survival (CSS) by clinically meaningful categories (NMIBC vs. MIBC) (**C**) and separately for Ta (**D**), T1 (**E**), and T2−4 (**F**) stage categories.

**Figure 5:**
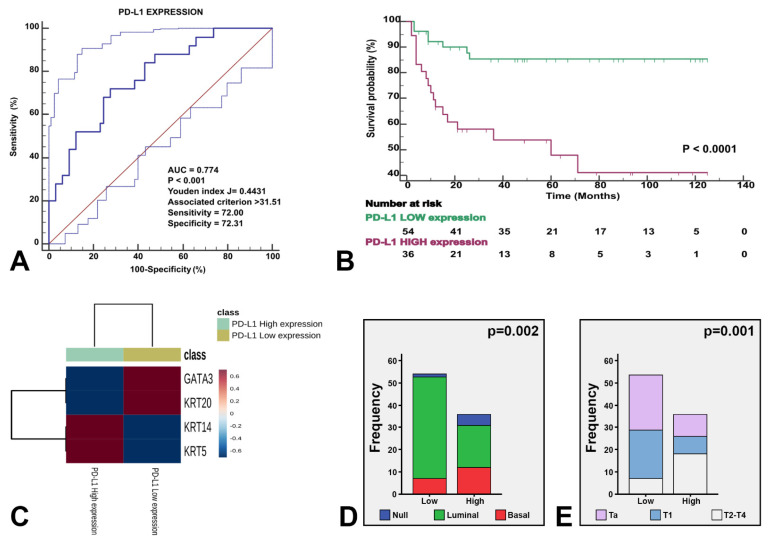
The three identified molecular subtypes were stratified by PD-L1 expression. Receiving operating characteristics yielded an AUC of 0.774 (*p* < 0.001) for the optimal cutoff point of PD-L1 expression (**A**), which illustrated survival differences in our study series (**B**). The heatmap shows the expression of the four genes stratified by high vs. low PD-L1 expression (**C**). The enclosed histograms depict differences between high vs. low PD-L1 expression according to molecular subtypes (**D**) and stage category (**E**).

NanoString-based gene expression analysis using markers typically considered luminal (GATA3+ and/or KRT20+) and basal (KRT14+ and/or KRT5+ and GATA3low/-/KRT20low/-) classified urothelial bladder carcinoma samples as luminal, basal and null/double negative (null/DN; non-luminal/non-basal), a third category (KRT14−/KRT5−/GATA3−/KRT20−). These three categories were meaningful in terms of CSS (Figure 3) and their associations to clinicopathological variables (Table 2), but also in terms of association to the clinical classification (NMIBC vs. MIBC) and AJCC stage categories (Figure 4), and PD-L1 correlations (Figure 5). A summary of the main characteristics of the three molecular subtypes is presented in Table 3. Table 4 illustrates a univariate and multivariate predictive model for CSS with model A incorporating Ta, T1 and T2-T4 AJCC categories, and model B incorporating T1 and T2-T4 AJCC categories. Interestingly, the analysis identified histological subtype, PD-L1 expression and molecular subtype as independent predictors of CSS, with higher values in model A.

## 4. Discussion

Studies have focused on developing a molecular classification potentially useful to predict prognosis and guide novel therapies in patients with bladder urothelial carcinoma to improve the current scientific knowledge of bladder cancer and provide a better framework for patient management [10,17,19,24,27,28,29,31,33,37,42,45,46,47,48,49,50,51,52,53,54,55,56].

During the last decade, several molecular classifications of urothelial bladder carcinomas have appeared. Following the major subtypes observed in breast carcinoma, these two categories were also recognized in urothelial carcinomas: luminal and basal molecular subtypes [13,28]. Interestingly, some of the reported classifications divided the luminal category into further subtypes; meanwhile, the basal subtype remained largely stable across the different classifications. For instance, Robertson et al. identified five categories (luminal-papillary, luminal-infiltrated, luminal, basal-squamous, and neuronal) to divide the luminal subtype into three additional categories [27]. Luminal categories were recently further delineated in the so-called consensus classification, which reported the luminal-papillary, luminal-unstable, and the luminal nonspecified [34]. Most of the reported molecular classifications to date incorporate the potential therapeutic implications associated with the reported category [20,37]. Then, the potential for fibroblast growth factor receptor 3 (FGFR3) inhibitors, low sensitivity to NAC, and variable ICI treatment response characterize the luminal subtypes. Cisplatin-based NAC, the potential for epidermal growth factor receptor (EGFR) inhibitors, and good response to ICI treatment characterize the basal molecular subtypes [37,54,55]. These molecular classifications originated from genomic and transcriptomic profiles that produced highly diverse classifications lacking any correlation between each other, a fact considered behind their limited clinical implementation. However, some improvements brought by the recently published consensus molecular classification of MIBC might change the landscape of molecular classifications of bladder cancer in the future [34].

To overcome the limitations associated with the complexity of the required technology, the high costs, and the limited availability of this technology worldwide, we conducted gene expression analysis using a four-gene panel typically related to luminal (GATA3+/KRT20+) or basal (KRT5+/KRT14+) based on NanoString technology and nCounter analysis in a series of 91 bladder cancer cases. This novel technology determined the molecular subtypes by mRNA expression of GATA3 or KRT20 for luminal (71%) and KRT5 or KRT14 for the basal (21%) subtypes. Similar to what was reported by other molecular classifications, our data provided different prognostic and therapeutic sensitivities associated with both major subtypes. Thus, consistent with low aggressiveness, the luminal molecular subtype was enriched in NMIBC with the morphology of conventional urothelial carcinoma, low PD-L1 expression, and low bladder cancer-related mortality. Conversely, consistent with high aggressiveness, the basal molecular subtype was enriched in pT2–4 disease with variant histology in patients who died of bladder cancer. Notably, this category was also enriched in high PD-L1 expression, opening an opportunity for these patients to be treated using ICI protocols [39,52]. A paradoxical situation is seen in MIBC basal molecular subtype since, as reported, it is a highly aggressive disease. Nonetheless, a better CSS than the luminal subtype can be achieved due to the good response to current therapies associated with the basal molecular subtype.

On the other hand, the lack of expression of the four markers allowed us to identify a third category, the null/DN subtype, in 8% of our cases. A similar category was also reported by Rebola et al. and Kim et al. using immunohistochemistry in NMIBC and MIBC, respectively [29,31,33,37,49]. A similar marker selection signature using immunohistochemistry was used to classify bladder cancer into luminal and basal categories with a high level of accuracy. However, the main limitation of the immunohistochemical method is the variability in the staining between samples across different institutions and that it is observer-dependent. A null/double negative category was also recently identified by Guo et al. analyzing mRNA expressions signature of luminal and basal that was used to develop a classifier with high sensitivity (80–94%) and specificity (83–93%) to identify molecular subtypes of bladder cancer [37]. In this study, GATA3/CK5-6 immunohistochemical signature was able to identify molecular subtypes with 80% accuracy. This study concluded that they had developed a tool for the assessment of molecular subtypes of bladder cancer in routine clinical practice [37].

In line with these results, our study further supports the feasibility of NanoString technology to provide a tool to accurately investigate the major molecular subtypes of urothelial bladder carcinoma using a relatively simplified four-gene expression panel with low cost and fast turnaround time. Another study in favor of this approach is the recent study that concurrently compared the so-called BASE47 genes in high-grade urothelial carcinoma using RNASeq and NanoString [35]. In this study, the classifier for luminal and basal molecular subtypes based on NanoString and nCounter analysis was validated in an independent dataset; the training and validation datasets accurately classified 87% and 93% of samples, respectively [35]. These results support luminal and basal molecular subtypes as clinically relevant categories when classified by NanoString methods, thus, providing a rationale for clinical application, as is the case of the Prosigna test, a NanoString-derived classifier currently in use to manage breast cancer patients [56]. Limitations of the current study include the retrospective nature and the relatively small sample size. Nonetheless, the long follow-up (median of 46 ± 40.51, 2–125 months) of our cases may add value to the current series.

## 5. Conclusions

In conclusion, using a simplified four-gene signature with NanoString nCounter assay provides a practical, cost-effective platform to translational research in the field of molecular taxonomy of bladder carcinoma, identifying three clinically meaningful molecular subtypes (luminal, basal, and null/double negative). Luminal tumors were associated with NMIBC with conventional urothelial carcinoma morphology, lower levels of PD-L1 expression, and favorable bladder-related survival. Conversely, basal and null/double negative molecular subtypes shared a higher frequency of MIBC enriched in variant histology, with high PD-L1 expression (likely to respond to ICI immunotherapy) and worse bladder cancer-related mortality.

## Figures and Tables

**Table 1:** Demography and clinicopathological characteristics of 91 bladder urothelial carcinomas included in the study.

Variables	N (%)
Age (median ± SD, range), in years	73 ± 10.25, 45–95
Gender	
Female	11 (12.1)
Male	80 (87.9)
* Stage category (%)	
Ta	36 (39.6)
T1	30 (32.9)
T2–4	25 (27.5)
Urothelial carcinoma categories	91 (100)
Conventional	67 (73.6)
Ta	36 (39.5)
T1	24 (26.4)
T2–4	7 (7.7)
Variant histology	24 (26.4)
Ta	-
T1	6 (6.6)
T2–4	18 (19.8)
Grade	
LG	27 (29.7)
HG	64 (70.3)
Risk categories NMIBC	
Low	14 (21.2)
Intermediate	13 (19.7)
High	33 (50)
Very High	6 (9.1)
Variant histology subtypes	
Micropapillary	6 (6.6)
Nested	6 (6.6)
Plasmacytoid	5 (5.5)
Other variants	7 (7.7)
Followup (median ± SD, range), in months	
Overall	46 ± 40.51, 2–125
Conventional urothelial carcinoma	49 ± 37.88, 2–125
Carcinoma with variant histology	9 ± 41.44, 2–122
Stage category	
Ta	78 ± 40.08, 8–125
T1	48.5 ± 33.69, 2–123
T2–4	9 ± 19.88, 2–90
Survival status (median ±SD, range), in months	
Alive	74 ± 36.78, 8–125
Cancer-specific survival	9.5 ± 17.30, 2–71

* Stage category based on AJCC/TNM 2016 revision.

**Table 2:** Relationship between molecular subtypes and clinicopathological parameters of 91 bladder carcinomas included in the study.

Clinicopathological Features	Overall n = 91 (100%)	Luminal n = 65 (%)	Basal n = 19 (%)	Null/DN n = 7 (%)	*p*-Value *
Survival status					<0.0001
Alive	34	32 (94.1)	1 (2.9)	1 (2.1)	
Alive with disease	3	0 (0)	3 (100)	0 (0)	
Dead bladder cancer	26	9 (34.6)	12 (46.2)	5 (19.2)	
Dead other causes	28	24 (85.7)	3 (10.7)	1 (3.6)	
Urothelial carcinomas					0.001
Conventional	67	55 (82.1)	8 (11.9)	4 (6)	
Micropapillary	6	4 (66.7)	2 (33.3)	0 (0)	
Nested	6	1 (16.7)	4 (66.7)	1 (16.7)	
Plasmacytoid	5	1 (20)	2 (40)	2 (40)	
Other variants	7	4 (57.1)	3 (42.9)	0 (0)	
Stage category					<0.0001
Ta	36	31 (86.1)	3 (8.3)	2 (5.6)	
T1	30	27 (90)	3 (10)	0 (0)	
T2-T4	25	7 (28)	13 (52)	5 (20)	
PD-L1 expression					0.002
High	36	19 (52.8)	12 (33.3)	5 (13.9)	
Low	54	46 (85.2)	7 (13)	1 (1.9)	

* Chi-squared test. Molecular subtype by NanoString. PD-L1 by RT-PCR. DN: Double negative. Stage category based on AJCC/TNM 2016 revision.

**Table 3:** Characteristics of the molecular subtypes using NanoStringnCounter analysis in the current study of 91 urothelial carcinoma (UC) samples.

Molecular Subtypes (n, %)	Histological Subtypes (n; %)	T Stage Category (n)	High PD-L1 Expression (%)	Survival Status, Median ±SD (Range), in Months
LUMINAL (65; 71%) GATA3^+^ and/or KRT20^+^	Conventional UC (55; 84.6%)	Ta (31); T1 (21); T2–T4 (3)	15 (27.3%)	49 ± 35.70 (12–125)
	Variant histology UC (10; 15.4%) (Micropapillary (4); Nested (1); Plasmacytoid (1); Other Variants (4))	Ta (0); T1 (6); T2–T4 (4)	4 (40%)	45 ± 55.36 (4–119)
BASAL (19; 21%) KRT5^+^ and/or KRT14^+^; GATA3^−^; KRT20^−^	Conventional UC (8; 42.1%)	Ta (3); T1 (3); T2–T4 (2)	4 (50%)	21 ± 7.34 (12–60)
	Variant histology UC (11; 57.9%) (Micropapillary (2); Nested (4); Plasmacytoid (2); Other Variants (3))	Ta (0), T1 (0); T2–T4 (11)	8 (72.7%)	9.5 ± 21.42 (2–21)
NULL/DOUBLE NEGATIVE (7; 8%) GATA3^−^; KRT20^−^; KRT5^−^; KRT14^−^	Conventional UC (4; 57.1%)	Ta (2); T1 (0); T2–T4 (2)	2 (66.7%)	32 ± 36.77 (6–58)
	Variant histology UC (3; 42.9%) (Nested (1); Plasmacytoid (2))	Ta (0); T1 (0); T2–T4 (3)	3 (100%)	4 ± 1.15 (2–4)

**Table 4:** Univariate and multivariate analysis of clinic-pathological parameters related to cancer-specific survival prediction in the current study.

Variable (Model A)		Univariate Analysis			Multivariate Analysis		
		HR	95% CI	*p* Value	HR	95% CI	*p* Value
Age, median, in years	≤73	1.389	0.642–3.004	0.405	-	-	ns
	>74						
Stage categories	Ta-T1	24.250	9.262–63.496	<0.0001	-	-	ns
	T2-T4						
Grade	LG	44.390	1.575–1250.955	0.026	-	-	ns
	HG						
Risk Categories	Low/Intermedium/High	2525.64	1.202–5,315,156	0.045	-	-	ns
	Very High						
Histological Subtypes	Conventional UC	6.660	3.013–14.721	<0.0001	3.825	1.590–9.202	0.003
	Variant Histology UC						
PD-L1 expression	Low	4.685	1.951–11.253	0.001	2.651	1.040–6.760	0.041
	High						
Molecular Subtypes	Luminal	7.124	3.148–16.124	<0.0001	3.870	1.570–9.541	0.003
	Basal/Null						
Variable (Model B)							
Age, median, in years	≤73	0.926	0.420–2.038	0.848	-	-	ns
	>74						
Stage categories	T1	11.792	4.265–32.602	<0.0001	-	-	ns
	T2-T4						
Grade	LG	20.958	0.000–2,048,943	0.604	-	-	ns
	HG						
Risk Categories	Low/Intermedium/High	241.387	5.392–10,806.678	0.005	-	-	ns
	Very High						
Histological Subtypes	Conventional UC	2.899	1.277–6.582	0.011	2.074	0.888–4.844	0.092
	Variant Histology UC						
PD-L1 expression	Low	3.537	1.471–8.505	0.005	2.267	0.890–5.772	0.086
	High						
Molecular Subtypes	Luminal	4.843	2.104–111.47	<0.0001	3.673	1.505–8.967	0.004
	Basal/Null						

HR Hazard Ratio; 95% CI 95% Confidence Interval. Model A incorporates Ta, T1 and T2-T4 AJCC categories; Model B incorporates T1 and T2-T4 AJCC categories.

## Data Availability

Data available on request due to privacy restrictions.
